# A multicentre survey on the perception of palliative care among health professionals working in haematology

**DOI:** 10.1007/s00520-024-08452-z

**Published:** 2024-03-27

**Authors:** Sara Di Lorenzo, Lisa Mozzi, Flavia Salmaso, Claudia Silvagni, Silvia Soffientini, Vanessa Valenti, Vittorina Zagonel

**Affiliations:** 1 Clinical Hematology and Bone Marrow Transplant and Cellular Therapies Center, Carlo Melzi”, Azienda Sanitaria Universitaria Friuli Centrale (ASU FC), Udine, Italy; 2 Clinical Hematology, Azienda Ospedaliera Ulss 8 Berica, “St. Bortolo” Hospital, Vicenza, Italy; 3 Palliative Care Unit, IRCCS Istituto Oncologico Veneto IOV, Padua, Italy; 4 Continuity of Care Center, Istituto Per La Sicurezza Sociale, Cailungo, Republic of San Marino; 5 Integrated Home Care Unit, AULLS 6 Euganea - Terme Colli District, Padua, Italy; 6 Palliative Care Unit, IRCCS Istituto Romagnolo Per Lo Studio Dei Tumori (IRST), “Dino Amadori”, Via P. Maroncelli 40, Meldola, FC 47014 Italy; 7 Medical Oncology Unit 1, Department of Clinical and Experimental Oncology, IRCCS Istituto Oncologico Veneto IOV, Padua, Italy

**Keywords:** Palliative care, Haematological malignancies, Perceptions of palliative care, Attitudes toward palliative care, Barriers to palliative care, Unmet palliative care needs

## Abstract

**Purpose:**

Patients with haematologic malignancies have less access to palliative care and are referred later than patients with solid tumours. We developed a survey to investigate this phenomenon, with the intention of analysing palliative care perceptions among health professionals who treat haematology patients and identifying barriers and facilitators to referrals to palliative care services.

**Methods:**

This was a multicentre exploratory descriptive web-based survey. A questionnaire was administered to 320 medical and nursing staff members from five Italian haematological units and San Marino’s hospital to investigate their perception of palliative care. Quantitative and qualitative analyses were performed.

**Results:**

A total of 142/320 healthcare professionals completed the survey, achieving a 44% response rate. Most of the respondents supported the integration of haematology and palliative care and were aware of the role of palliative care. Despite this, only half had an in-hospital palliative care team, and only a few had previously attended a specific training course. The majority agreed with palliative care referral when the prognosis was less than 3 months or when the symptoms were incoercible and with blood transfusions even in the last stages of the disease. Many considered the presence of an in-hospital palliative care team or a case manager, as well as structured palliative care training, as fundamental facilitators of palliative care referrals.

**Conclusion:**

These results showed that healthcare professionals in haematology generally hold a favourable attitude and a high interest in integrating palliative care into their patients’ care. The low referral rate could depend on clinical, cultural, and organisational issues.

## Introduction

Haematological malignancies (leukaemia, lymphoma, and myeloma) all have different aetiologies, prognoses, and frequencies [1]. According to data provided by the “Global Cancer Observatory” in 2020, the diagnosis of haematological neoplasms corresponds to 6.64% of all cancer diagnoses, with an overall mortality of 7.13% [2]. These illnesses are characterised by long and complex prognosis, unpredictable disease trajectories, rapid clinical deterioration, and high symptom burden due to polychemotherapy regimens, radiotherapy, and/or bone marrow transplantation [1, 3, 4]. Urgent hospitalisations for serious medical complications are frequent, especially in the advanced stage of the disease. However, there is a growing availability of new treatments that contribute to increasing both the possibility of recovery and long-term survival [1, 4]. This can affect patients’ quality of life, particularly in cases of very long hospitalisations or intensive medical treatments up to the last stages of life. Recent international literature supports the integration of palliative care (PC) and haematology with improved outcomes, particularly in models of early integration and simultaneous care [5–7], where supportive care does not exclude active treatments and collaboration between professionals is structured throughout the patient’s care pathway, pursuant to emerging needs. These models have been shown to promote higher-quality symptom management, facilitate complex medical decision-making, contribute to reducing hospitalisations and intensive medical treatments with an adverse harm/benefit ratio, and lower healthcare costs [5, 6, 8]. Despite recent evidence, fewer haematologic patients access PC services compared to patients with solid cancers [1, 4, 9, 10]. The reasons for this phenomenon include cultural aspects, attitudes that propagate during medical training, the unique nature of haematological malignancies, such as difficulty with prognostication, and lack of accessibility to PC services.

These barriers cross a variety of cultural contexts, which highlight the broad scope of the problem and emphasise the need for durable and sustainable solutions [1, 2, 4, 10–20].

In Italy, only one study has previously analysed the cognitive barriers and facilitators of health professionals when referring patients to PC via a qualitative survey [15]. Only two other studies support the early integration of PC and haematology, and both demonstrated the effectiveness of these models in improving quality of life and significantly reducing healthcare costs [21, 22]. This study aimed to investigate the barriers and facilitators perceived by haematologic healthcare professionals in referring patients to PC and to propose a variety of solutions to improve collaboration between palliative and haematologic care.

## Materials and method

### Study procedures

The study was formally notified to the Ethics Committee of the Istituto Oncologico Veneto of Padova, and the health departments of each centre gave their approval. This research is a web-based, multicentre, exploratory descriptive survey. Eligible participants were specialist and trainee physicians as well as nurses working at an onco-haematological inpatient or day hospital of five Italian haematological units and San Marino’s hospital, specifically: IRCCS - Istituto Oncologico Veneto (IOV) of Padova and Castelfranco Veneto, Azienda Ospedaliera di Padova, Azienda Ospedaliera of Vicenza, Azienda Sanitaria Universitaria Friuli Centrale (ASU FC) of Udine, IRCCS-Istituto Romagnolo per lo Studio dei Tumori “Dino Amadori” (IRST) of Meldola, and Istituto per la Sicurezza Sociale (ISS) of San Marino.

This study arose from our direct experience of resistance by haematologists to referring patients to palliative care. We confirmed, through a review of the literature, that the identical issues we noted had emerged in other settings formatively and culturally distinct from ours. Considering that no study has been conducted in Italy, we proposed a multicentre survey to promote awareness among health professionals about this topic in the hopes that further investigations will be conducted. This is why we decided to limit participation to academic healthcare professionals: we want this survey to serve as a starting point for future studies that will promote synergy between PC and haematologic care, while also including the public and raising awareness.

Participants were enrolled via an email invitation that explained the purpose of the study and included a link to complete the questionnaire on the Google Forms digital platform.

### Study measures

The initial stage in creating the questionnaire was to conduct a non-systematic review of the literature on PubMed, Cochrane, Chinal, and Scopus using the following keywords: palliative care, barriers, onco-haematology, haematological malignancies, hospice, end-of-life, interposed by Boolean operators “and”, “or”. Articles published prior to 2010 were excluded, to have recent and up-to-date data and a context more representative of the current reality. The questionnaire was developed using the collected bibliography and tailored for distribution to medical professionals and nurses under the supervision of a qualitative research expert. Validation was not necessary because the project consisted of a survey sent to healthcare experts rather than a measurement scale. Nonetheless, as a model, we used a similar questionnaire previously administered to transplant physicians in the USA [16].

The questionnaire was created using the Google Forms digital platform, as it is a safe and secure tool, widely used for this type of exploratory investigation. Professionals could participate anonymously, and the compilation process took an estimated 20 min overall. The following areas were investigated:

- Personal information and clinical practice characteristics (6 items)
- Knowledge of PC (4 items)
- Education and training in PC (1 item)
- Perceptions of professionals regarding facilitators and barriers (8 items)
- Personal experiences (2 short open-ended questions)
- The questionnaire was administered over the course of 20 days, in September and October 2021.

### Statistical analysis

The study’s objectives are descriptive: for the quantitative and qualitative closed-response variables, statistical analyses were carried out using SAS software. After the data were described, the frequency and response rates were correlated with age and profession status according to multivariate analysis.

Microsoft Excel software was used to categorise the open-ended responses, which were then examined separately through group discussion. Labels were applied to identify recurrent thematic areas and relative intensity; significant responses were fully reported to support the discussion.

The questionnaire responses and the associated raw data are available and can be consulted upon request by the authors.

## Results

### Participant characteristics

Table 1 lists the characteristics of the participants. Of the 320 health professionals involved, 142 (44.4%) answered the questionnaire.

**Table 1:** Participant characteristics (*n* = 142)

	*n* (%)				
Age					
20–30	36 (25.4)				
31–40	53 (37.3)				
41–50	36 (25.4)				
51–60	17 (12)				
> 60	0				
Sex					
Female	105 (73.9)				
Male	36 (25.4)				
Nonbinary	1 (0.7)				
Years of Clinical Practice in Haematology					
< 1	15 (10.6)				
1–5	51 (35.9)				
6–10	29 (20.4)				
11–15	14 (9.9)				
16–20	17 (12)				
21–25	12 (8.5)				
> 25	4 (2.8)				
Profession					
Physician	46 (32.4)				
Nurse	96 (67.6)				
Medical institution					
Istituti di Ricovero e Cura a Carattere Scientifico (IRCCS)	42 (29.6)				
Hospital	52 (36.6)				
University Hospital	48 (33.8)				
Access to palliative care service and training					
Presence of the palliative care team in their own centre	72 (50.7)				
Professionals who have never participated in palliative care training courses	77 (54.2)				
Professionals who have participated in palliative care training courses outside their centre	42 (29.6)				
Professionals who have participated in palliative care training courses inside their centre	23 (16.2)				

With a fairly homogeneous distribution concerning working reality, nurses (96/142, 67.6%) made up the majority of the sample. Just over half of the interviewees (72/142, 50.7%) reported the presence of an in-hospital PC team, and 77/142 (54.2%) never attended PC-related courses.

### Knowledge and perceptions of palliative care

Most participants (100/142, 70.4%) stated that they knew the role of PC: when asked to supply a list of keywords that could be used to define PC, (119/142, 83.8%) answered. Every response was examined, categorised into macro-areas, and broken down into a total of 321 keywords. The most prevalent categories were “end-of-life and death” (37/321, 11.5%), “accompaniment” (42/321, 13.1%), “support” (28/321, 8.7%), “global care” (29/321, 9%), “symptoms” (44/321, 13.7%), and “quality” (73/321, 22.8%).

**Fig. 1:**
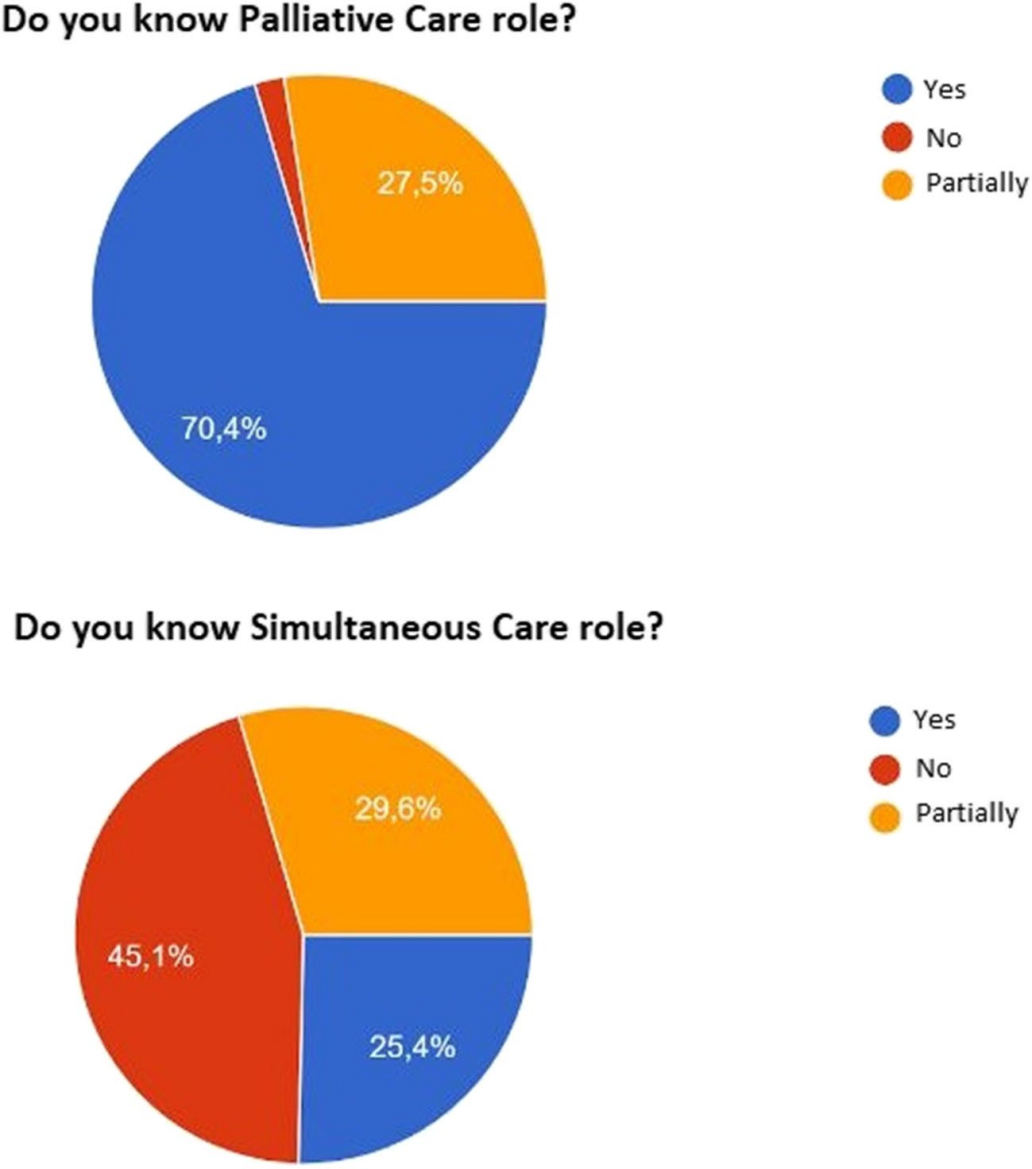
Participants’ knowledge of palliative care and the simultaneous care role

Concerning simultaneous care, 45.07% (64/142) of participants said that they were unaware of the role it plays (Figure 1). We again asked the participants to supply a list of keywords that could be used to define simultaneous care: (110/142, 77.5%) answered. Every response was examined and categorised into macro-areas like: “integration and multidisciplinary team” (32/110, 29.1%), “early management and timing” (27/110, 24.5%), “symptoms, pain, and side effects of therapies” (18/110, 16.4%), and “globality” (8/110, 7.3%).

Subsequently, 113/142 (79.6%) of the medical professionals involved strongly agreed with the statement that PC integration in haematology benefits patients and caregivers.

**Fig. 2:**
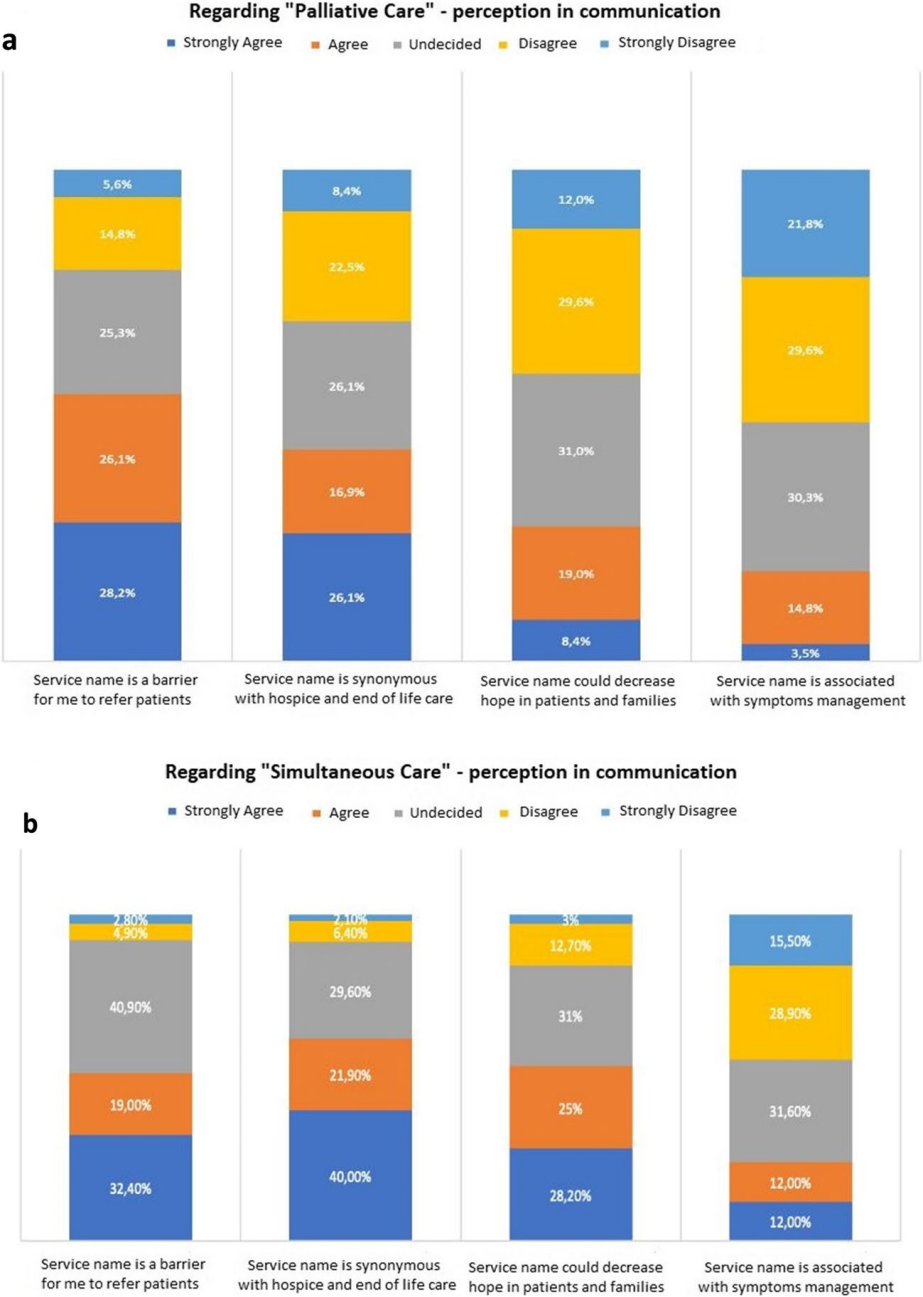
Participants’ perspectives on the terms “palliative care” (**A**) and “simultaneous care” (**B**)

At this point, we examined how the terms “simultaneous care” and “palliative care” affected dialogue (Fig. 2).

First, we asked participants if they thought the terms “palliative care” and “simultaneous care” would prevent PC referrals. Overall, 77/142 (54.3%) answered affirmatively for “palliative care”, and 73/142 (51.4%) for “simultaneous care”. “Hospice” and “end of life” are synonymous terms for 61/142 (43%) of “palliative care” and 88/142 (61.9%) of “simultaneous care”. When asked if the term “palliative care” could make patients and caregivers feel less hopeful, only 39/142 (27.4%) participants said they thought so; however, 76/142 (53.5%) said they thought the same about “simultaneous care”. A few of the interviewees (26/142, (18.3%) agreed that the term “palliative care” could be linked to the management and treatment of exclusive symptoms, while 34/142 (24%) were more concerned with “simultaneous care”.

### Access to palliative care services

**Fig. 3:**
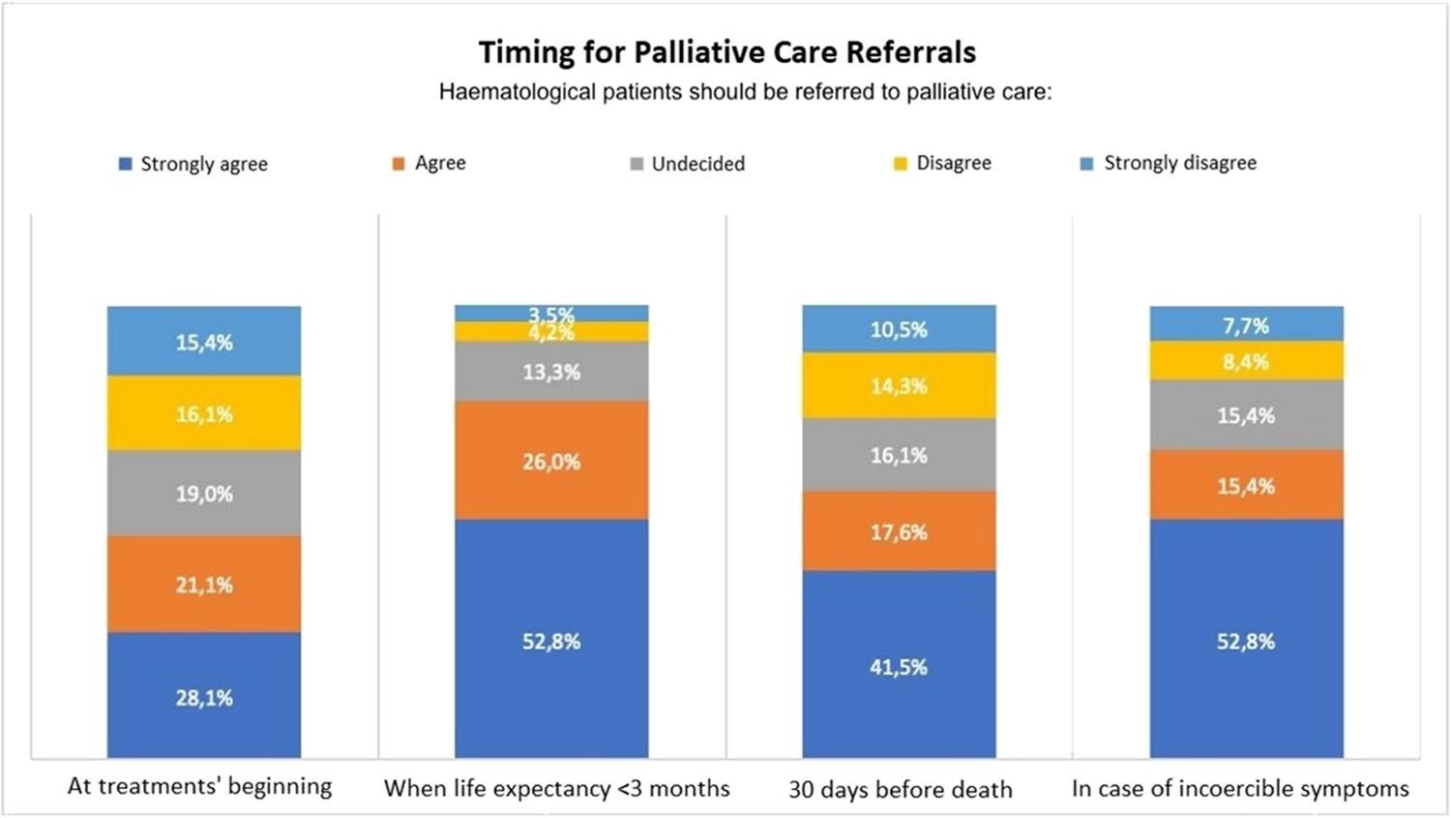
Professionals’ perspectives on the timing of palliative care referrals for haematological patients

The timing of the PC referrals was then examined (Figure 3). A small majority of participants (75/142, 52.8%) strongly agreed to request PC when the prognosis was less than 3 months, while only 70/142 (49.2%) agreed to do so for haematological patients at the start of treatment. On referrals made 30 days prior to death, 84/142 (59.1%) agreed. On the other hand, there is a greater consensus (97/142, 68.2%) regarding PC referrals when symptoms become unmanageable.

**Fig. 4:**
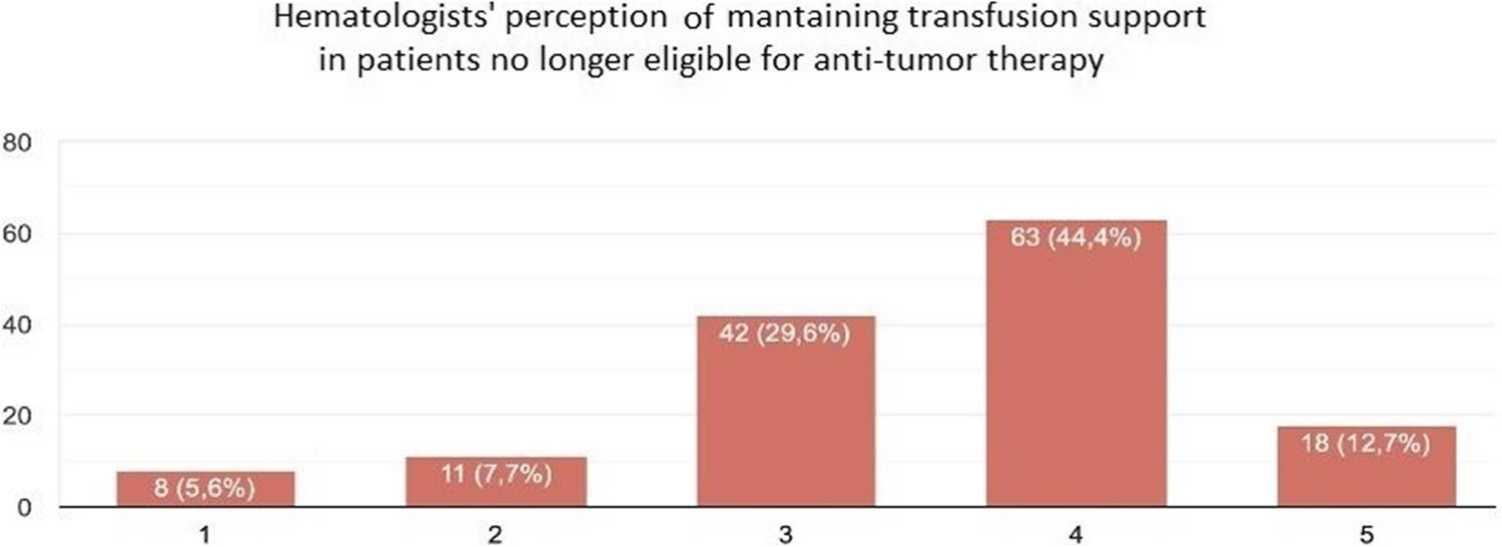
Haematologists and nurses’ perceptions of maintaining transfusion support in patients no longer eligible for antitumour therapy. A 5-point Likert scale was used for rating professionals’ perceptions of transfusion support: scale (1) was assigned for total agreement with the statement and (5) for complete disagreement

Figure 4 shows the perception of maintaining transfusion support in patients no longer eligible for antitumour therapy; this statement was supported by the majority of participants (68/142, 57.1%). Of those, 18/142 (12.7%) fully agreed and 63/142 (44.4%) agreed.

Through a correlation analysis between the participants’ profession and these data, it was possible to determine that, on average, physicians are more favourable (24/46, 60.9%) than nurses are (53/96, 55.3%), with most nurses not taking a position (31/96, 32.3% compared to 11/46, 23.9% of doctors).

In response to the open-ended question about health professionals’ opinions concerning referral to the PC team, 122/142 (85.9%) answered, and 111/122 (90.9%) responded positively. The analysis was performed again for these open-ended responses by dividing the 161 recurring keywords into 8 macro-areas: “opportunity for the patient” (48/161, 29.8%), “quality of life and dignity” (39/161, 24.2%), “multidisciplinarity” (21/161, 13%), “total care” (20/161, 12.4%), “grief awareness and processing” (15/161, 9.3%), and “support” (9/161, 5.6%). Merely 4.9% (6/122) expressed dissent, emphasising that the primary issues stemmed from the demoralisation of patients and caregivers, an inadequate PC network in fulfilling patients’ requirements, and haematologists who view PC referrals as a personal failure.

Consequently, we tried to delve deeper into two significant and related areas. First, the reasons behind the PC’s request for intervention were discussed: 105/152 (74%) fully agreed with the clinical aspects; 112/142 (78.8%) agreed with the communicative-relational reasons; 107/142 (75.4%) agreed with the ethical and deontological aspects; and 116/142 (81.7%) agreed with the management of emotional load.

The second part involved who should communicate the prognosis in an advanced stage of illness by referring the patient to the PC team. The first hypothesis was that the haematologist would communicate the prognosis; 39/142 (27.5%) participants strongly agreed, and 53/142 (37.3%) participants agreed. The second involved the conjunction between haematologist and the palliative specialist; 101/142 (74.2%) participants agreed. The final one suggested that the multi-professional team communicate it; 70.4% (100/142) of the participants strongly agreed, and 15.5% (22/142) of participants agreed.

Lastly, we used an open-ended question to investigate circumstances in which professionals might have considered it appropriate to refer patients to PC services but chose not to, and if that was the case, we asked why: 110/142 (77.5%) participants confirmed the occurrence of this eventuality. We classified 150 reasons that prevented access to the PC into recurrent thematic areas by analysing the affirmative answers: “professionals” perceptions” (23/150, 33%), “professional training and experience” (34/150, 22.66%), “institute resources” (24/150, 16%), “prognostic timing” (21/150, 14%), “patients” and caregivers” altered perceptions and awareness” (15/150, 10%), “lack of professional collaboration” (13/150, 8.67%), “patients” persistence in therapy” (13/150, 8.67%), and “the doctor-patient relationship” (7/150, 4.67%).

### Perceived facilitators to palliative care utilisations

**Fig. 5:**
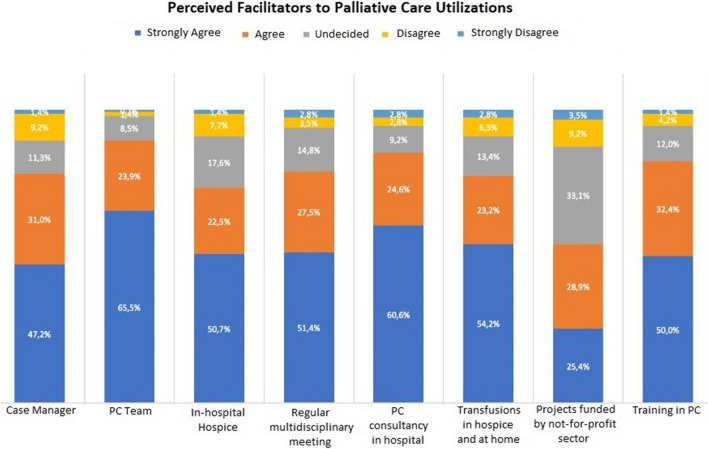
Professionals’ perspectives on possible facilitators of palliative care utilisation

Figure 5 outlines participant perceptions of elements that might encourage PC team referrals. Having a dedicated case manager is the first potential facilitator that has been explored. Most professionals view this as a factor that favours consistent exchange with the palliative team: 44/142 (31%) agreed, and 67/142 (47.2%) completely agreed. The PC team’s presence within the hospital is yet another suggested facilitator, with 89.5% (135/142) of consent; specifically, 42/142 (24%) respondents agreed and 93/142 (65.5%) strongly agreed.

Participants rated the availability of an in-hospital hospice as a facilitator in 73.2% (104/142) of cases. Similarly, 73/142 (51.4%) strongly agreed, and 39/142 (27.5%) agreed that regular meetings with a PC team could enhance the integration with haematologists.

Regarding the option to request an in-hospital palliative consultation, 86/142 (60.6%) of the respondents strongly agreed that it is a process facilitator. Moreover, most health professionals (110/142, 77.5%) believed that the ability to transfuse patients in hospice or at home could be a facilitating factor. Ultimately, the majority of them viewed training programmes as a motivating factor for patients to be referred to PC (71/142, 50% in strong agreement and 46/142, 32.4% in agreement).

## Discussion

This is the first study conducted in Italy with the goal of thoroughly examining how medical professionals who work in onco-haematology departments feel about PCs.

The integration of PC and haematology benefits patients and healthcare professionals according to 96.5% (137/142) of participants (113/142, 79.6% strongly agreed, and 24/142, 16.9% agreed). However, one of the most identified barriers is the lack of services, such as an in-hospital PC team, which is crucial for determining which patients can benefit from the service, and an in-hospital hospice.

Transfusion support is a highly debated topic in the care of haematological patients, as it is frequently a binary decision for PC referrals [12].

According to the gathered data, transfusion support is a critical component for off-therapy patients. As a result, it is thought that offering transfusions at home and in hospice settings encourages PC team referrals, particularly from physicians. Despite the fact that healthcare professionals have written extensively about this subject in the literature [23–27], clinical practice still lacks a formalised, shared, and comprehensive process to support decision-making and encourage communication between two teams.

This survey highlights the issue of professional collaboration as a barrier to PC referrals. According to the literature [6, 17], multidisciplinarity seems to help lower cultural barriers related to the role of individual professionals. Nurses were more aware of this issue (82/96, 84.3%), and it is evident that while haematologists understood the value of integrating with the PC team, the volume of referrals did not match the needs that were identified.

A significant disparity between areas where equitable, accessible, and continuous care is not guaranteed is caused by the lack of uniform organisational models and care pathways for patients with PC needs. Integrated care models [5–8] encourage increased collaboration and communication between PC and haematology providers during disease treatment [7, 16, 28].

A facilitating factor that is still lacking in regulatory identity but acknowledged by 112/142 (79%) of the involved professionals is the case manager nurse, who establishes goals, maintains consistency across various care settings, and recognises interdisciplinary issues.

Although there are many resources available to improve the standard of care, training and education remain among the easiest to apply. According to the study, 100 professionals were familiar with PC but had never taken any related courses. They cited several factors, including lack of experience, inadequate training, and mistrust of haematologists, as contributing to unsuccessful PC referrals.

Specific training focusing on the complexity of needs is necessary for PC diffusion in various care settings, but it is still inadequate in university education and in the general culture [29, 30]. Even though PC has gained recognition as a medical specialty and that many professionals and students acknowledge its benefits, university curricula still frequently omit PC courses [30].

Note that there is a lack of current literature on PC training and education programmes, as well as on the specific role of the case manager; of the studies we found, only two were published after 2018 [20, 29].

Due to cultural barriers and misconceptions, haematologists and palliativists do not work together as much as they should. Based on the data gathered, however, university education and public awareness campaigns might reduce PC deficiency.

The timing of PC referral appears to be another significant concern in this study and a prominent theme in the literature [4, 11, 28, 31]. Early referral of haematological patients to PC is hindered by the unpredictable nature of the haematologic disease trajectory, the timing of the treatments, and the possible complications. It is helpful to highlight professional differences, such as the fact that some doctors (15/142, 10.56%) and nurses (12/142, 8%) disagreed with administering on PC at the start of treatment.

In haematology, a PC model is still lacking today [4], and regardless of the patient’s anticipated life expectancy, when to activate PC is crucial.

The literature contains evidence that a shared path between PC and aggressive curative treatment (such as conditioning chemotherapy and related transplantation) is possible at the same time, accepted by patients, and has positive outcomes [32, 33]. An outpatient observational study of patients with acute myeloid leukaemia revealed that those who received early palliative supportive care had a greater quality of life and lower rates of treatment aggressiveness at the end of life [34].

The survey’s results are strikingly similar to those in the literature, which seems to be mostly composed of European and international sources [1, 13, 15, 19, 35].

Even though they are familiar with PC, the majority of the haematologists involved do not think that they are essential, so the service is only activated a few days prior to death, just as they are not aware of the role of simultaneous care (64/142, 45.07%); literature suggests that this model is still often a prerogative of oncology [9, 13, 15].

According to the survey, one significant perceived barrier is the use of the terminology used when proposing the service to patients and family members: healthcare professionals did not acknowledge PC as an identity and are unable to discuss end-of-life because of the exclusive relationship established with patients.

In addition to the study’s identified barriers—which can be summed up as clinical, cultural, educational, organisational, and resource allocation—haematologic patients have access to a variety of therapeutic options that unavoidably postpone suspending active treatment. This has led medical professionals to speak about aggressive medical treatments in certain situations (13/142 answers).

The attitudes of the health professionals who participated in the survey generally support suggestions for integrating PC in haematology, indicating a need for improvement. Furthermore, there were no notable distinctions between the replies of doctors and nurses, demonstrating that both groups had similar understandings of the primary problems that surfaced.

Proactive suggestions for enhancement, such as case managers, shared procedures and protocol drafting [27, 36], integrated care models [6, 9, 11, 28], training [20], and population involvement, could help PC approach haematology in a way that guarantees the highest quality of life for patients and families.

## Conclusion

The peculiarities of haematological malignancies (unpredictable illness trajectory, elevated symptom burden, specific care needs), healthcare organisation models (presence of in-hospital PC teams and PC case managers, presence of integrated PC networks between hospital and territory, accessibility to palliative transfusions and chemotherapies), and cultural aspects (training and perceptions of professionals working in haematology, education, and development of shared care plans with caregivers) are, in summary, the main obstacles to haematological patients’ referral to PC units.

Certain suggestions were proposed to close the gap between these two disciplines based on the comparison of the literature and the data collected.

For example:

- Implementing an in-hospital PC team.
- A fully staffed service is frequently impractical due to limited human resources and healthcare policies. Instead, it would be appropriate to reorganise PC services that are currently in place and primarily involved in home and hospice settings and thereby ensures that the PC team has scheduled intrahospital access (e.g., once or twice a week), which would facilitate requests for consultation and be handled by haematologists.
-To promote the establishment of specialist outpatient clinics for PC and simultaneous care in haematology wards or day hospitals.
-To hold focus groups involving haematologists and palliative physicians to develop shared checklists that identify key indicators and the best time to refer these patients to PC services.
-To develop mutual guidelines and logistic context-specific procedures for transfusion support to guide physicians in decision-making.
- Expert talks or focus groups involving all professionals (transfusion medicine doctors, haematologists, and palliative physicians) could be used to build the former. The development of appropriate transfusion criteria could be accomplished using a checklist that considers blood values, the risk/benefit ratio (circulatory overload brought on by transfusion versus the reduction of anaemia symptoms), and the accessibility of substitute therapies such as iron infusion [24, 27]. To overcome organisational and resource barriers that prevent blood from being supplied to hospices or through home transfusion arrangements, logistical procedures should be developed by all parties involved in the pathway (hospital, territory, transfusion centre) [26, 27].
-To encourage collaborative staff training through hospital-specific courses, such as those offered as part of the mandatory yearly formation plan or new hire orientation.
-To provide haematologists with a training period in PC wards during their university specialisation, as does the training of palliative specialists, to improve their knowledge in both fields.
-Promote the presence of case managers.
- To enhance the integration between the two disciplines, it would be beneficial to have at least one member of the haematology team serve as an activity and services coordinator, and as a liaison between the patient, the healthcare system, and community resources. One of the primary objectives of the case manager is to lessen the patient's psychological distress and manage symptoms resulting from illness or treatment, thereby improving the quality of care for the patient and family. For this reason, this specialist may be the most important member of the haematologist team in determining the appropriate and pertinent palliative care referral.
- Following the identification of the need for PC, the case manager might encourage the haematologist to request consultation with a PC specialist or may suggest clinical cases for discussion via a multidisciplinary briefing between the two services.
- Furthermore, patients with haematologic malignancies require extensive clinical and logistic information to make treatment and clinical decisions, and case managers are experts in building consensus and empowering: they could present palliative care as one of the services and resources patients and families could access at anytime throughout their care path.
- To encourage the scheduling of multidisciplinary meetings, address urgent cases, and assess potential simultaneous care pathways.
- This survey has several limitations. The ability to complete the questionnaire exclusively online and performance bias—because participants were acquainted with the researchers—may have had an impact on the response rate. There is also respondent selection bias: the characteristics of nonrespondents were not collected, which could limit the ability to generalise the data findings; additionally, the study only examined the opinions of medical professionals who work with haematology patients, and the centres involved differed.

Future research should assess the viability and dependability of the suggested implementation pathways, explore in greater detail the variations among specific centres and explore the perspectives of palliative physicians. This approach allows the data to be cross-referenced with the actual numbers of patients who are sent to the PC.

## Data Availability

The data that support the findings of this study are not openly available due to reasons of sensitivity and are available from the corresponding author upon reasonable request. Data are located in controlled access data storage at IRCCS Istituto Romagnolo per lo Studio dei Tumori (IRST) “Dino Amadori”.
