# Correction to: A multicentre survey on the perception of palliative care among health professionals working in haematology

**DOI:** 10.1007/s00520-024-08699-6

**Published:** 2024-07-02

**Authors:** Sara Di Lorenzo, Lisa Mozzi, Flavia Salmaso, Claudia Silvagni, Silvia Soffientini, Vanessa Valenti, Vittorina Zagonel

**Affiliations:** 1 Clinical Hematology and Bone Marrow Transplant and Cellular Therapies Center, Carlo Melzi”, Azienda Sanitaria Universitaria Friuli Centrale (ASU FC), Udine, Italy; 2 Clinical Hematology, Azienda Ospedaliera Ulss 8 Berica, “St. Bortolo” Hospital, Vicenza, Italy; 3 Palliative Care Unit, IRCCS Istituto Oncologico Veneto IOV, Padua, Italy; 4 Continuity of Care Center, Istituto Per La Sicurezza Sociale, Cailungo, Republic of San Marino; 5 Integrated Home Care Unit, AULLS 6 Euganea - Terme Colli District, Padua, Italy; 6 Palliative Care Unit, IRCCS Istituto Romagnolo Per Lo Studio Dei Tumori (IRST), Dino Amadori”, Via P. Maroncelli 40, 47014 Meldola, FC Italy; 7 Medical Oncology Unit 1, Department of Clinical and Experimental Oncology, IRCCS Istituto Oncologico Veneto IOV, Padua, Italy

**Correction to: Supportive Care in Cancer (2024) 32:253**

10.1007/s00520-024-08452-z

The correct acknowledgment should be the below:

"This work is supported by 5x1000 anno di riferimento 2019 – Translational oncology: a multi-span bridge from discovery through development to implementation and effectiveness (BRIDGE).

The authors would like to thank all the study participants.

We would especially like to thank ASMEPA (Accademia delle Scienze di Medicina Palliativa), the professors, our supervisor, and all those who contributed to our ideas becoming a structured project that have helped us to examine a topic that is still little explored. We hope that this will motivate further studies and conversations."

And not

The authors would like to thank all the study participants.

We would especially like to thank ASMEPA (Accademia delle Scienze di Medicina Palliativa), the professors, our supervisor, and all those who contributed to our ideas becoming a structured project that have helped us to examine a topic that is still little explored. We hope that this will motivate further studies and conversations.

